# The Expansion Mechanism of the Cooperative Networks of Supply Support Organizations in a Public Health Emergency

**DOI:** 10.3390/healthcare9081041

**Published:** 2021-08-13

**Authors:** Chenxi Lian, Jian Wang, Jida Liu

**Affiliations:** School of Management, Harbin Institute of Technology, Harbin 150001, China; chenxi_lian@163.com (C.L.); kittadada@yeah.net (J.L.)

**Keywords:** cooperative network, public health emergency, exponential random graph model, expansion mechanism, emergency supply support

## Abstract

The outbreak of COVID-19 has significantly restricted the productive capacity of society and resulted in a shortage of supplies to maintain survival. Lightening the burden not only depends on government agencies, but also needs extensive social organization participation. However, few studies focus on how to promote social cooperation to support the provision of emergency supplies. This study aimed to find out the theoretical mechanism to expand the cooperative networks of supply support organizations during the epidemic. Data from the emergency response to the COVID-19 pandemic in China were used. Three cooperative networks from a progressive perspective were constructed based on the cooperative relationships among organizations. The expansion mechanism was verified by the exponential random graph model. The results show that when the institutional network expands into an interactive network, the composition of organization types has changed, but the cooperative network’s efficiency does not improve much. The matching effect of the organizational type and the Matthew effect of nodes are both effective paths to promote cooperative network expansion, however, the structure effect shows that complex relationship structure is not a critical factor. Our findings highlight the importance of core organizations and the function of different types of organizations in building cooperative network as well as providing theoretical frameworks for policymakers to use in guiding and motivating social cooperation in emergency supplies.

## 1. Introduction

The extensive global outbreak of COVID-19 has led to severe shortages of basic supplies. The World Health Organization reported that nearly a quarter of the world’s population was under quarantine, and markets for basic supplies and key medical supplies had contracted quickly by the end of March 2020. Due to strict specifications and a limited number of manufacturers and suppliers, it is complicated to produce a large amount of supplies in a short period of time. The Covid-19 Supply Chain System (CSCS) was built to bring together UN agencies, public health partners, suppliers and NGOs to provide material support to troubled areas. The emergence of a major public health issue is one of the greatest threats to social development, and supply support is a vital task for the government to deal with hazards and maintain people’s livelihoods [1]. To meet the demand for material in a major public health emergency, it is necessary to incorporate an extensive socialized emergency supply support beyond institutionalized and planned organizational support in a country or a region [2,3].

The production, procurement, delivery, and distribution of emergency supplies [4] as well as other steps, are interrelated and interlinked, making emergency supply support a complex system engineering issue. Additionally, the urgency [5], managing complexity [6,7], cross-jurisdiction [6,8], organizational diversity [9] and other risk characteristics of emergencies pose great challenges for emergency supply work. In existing theoretical studies, scholars believe that cooperative networks are organizational relationship systems for emergency response [10,11] and that they provide a structural frame for social capital. Scholars highlight the governance challenges across jurisdictions, levels, and borders [9,12] while accommodating the diversity of organizational participation. Studies have shown that the social resources provided by nongovernmental organizations, noncontracted businesses and individual donors have played an important role in large-scale emergency supply support [1,13]. Therefore, bringing these organizations into the cooperative network of emergency supplies is conducive to dealing with a severe and long-term public health crisis event [14]. The structural characteristics, overall performance, organizational role alignment and functional orientation of emergency management networks composed of government agencies have been explored. Some scholars have gradually begun to focus on the differences between planning networks and actual networks [15] and have attempted to build a widely socialized emergency organizational network. In short, emergency management networks are becoming more sophisticated. However, such networks are not yet sufficiently mature to deal with the problem of broad social cooperation. This situation underlines the rationality and urgency of our research, which probes the expansion mechanism of the cooperative networks of supply support organizations in major public health emergencies.

The expansion of such networks explains the path and mechanism by which multiple organizations are effectively incorporated into the emergency organization system from the perspective of network actors. However, directly merging and constructing a cooperative network will ignore the essential differences between institutionalization and socialization in cooperative relationships. Therefore, by adopting a progressive perspective from institutionalization to socialization, this paper develops three cooperative networks of institutional, interactive and social emergency supply support organizations. Taking the COVID-19 epidemic situation in China as a study scenario, this paper constructs an exponential random graph model (ERGM) to discuss the expansion mechanism of networks to wider social cooperation in an attempt to provide theoretical assistance for emergency supply support activities and the accumulation of emergency social capital. This paper makes the following contributions. First, three emergency networks are constructed with the idea of continuous integration and superposition, and the socialization characteristics of cooperation for emergency supply support are explained. Second, based on the embeddedness of social capital, this paper presents a comparative analysis of the network structure of the three networks above. Third, this paper proposes an expansion mechanism of networks for emergency supply support in the process of socialization, providing a theoretical supplement to policy making.

This paper is organized as follows: the second section collates and summarizes previous studies as the theoretical background. The third section proposes the research framework and describes the data and methods. Next, network analysis and model calculation are conducted, and the research results are presented. The results of the model are discussed considering the reality of emergency management. The last section presents our conclusions.

## 2. Literature Review

Since the 1980s, human beings have moved irreversibly into a risk society. Many types of risks and crises occur frequently, such as earthquakes, tsunamis and epidemics. Emergencies are unpredictable [9]. Preparation is necessary, but it is never possible to adequately prepare, making emergency organizing very difficult.

### 2.1. The Expansion of Participants in Emergency Management

Traditional emergency management organizations are mainly composed of government agencies and authoritative organizations, which establish connections and communication rules through planning policies [16,17]. Existing studies have shown that such institutionalized organizations can act rapidly and have made great efforts in the preparation, response, disposal and recovery process of emergency management [14]. However, it is still difficult to cope with large-scale and long-term crisis events. To obtain wider emergency support, research has considered the participation of more social organizations [13]. It is difficult for institutionalization to fully guarantee the effective participation of diversified social organizations and private enterprises. It is necessary to build social capital links with cooperative elements such as trust, common goals and technology utilization [11,18]. Additionally, social media have proven to be an essential supplement to emergency organizations [19]. Private organizations, institutions and even individuals on Internet platforms are considered a source of power for emergency management [19,20]. Nevertheless, such organizations are still at the periphery of the emergency organization system, while government agencies remain the core [21].

Supply chain research can be regarded as a perspective of emergency organization expansion. Singh et al. focused on the issue of supply chain disruptions for emergency supplies, suggesting that during a pandemic, essential items include food and medical equipment and that a flexible food and medical supply chain is needed [22]. Their study points to increased difficulties in matching supply and demand in an extensive public distribution network due to the growth of infections and changes in recovery. The COVID-19 outbreak is causing some concern because of the severe impact it has had on supply chains around the world [23], particularly in the area of medical services and health care, which is often the area where the need for specialized supplies is greatest in emergency management [24,25]. Martins et al. used a multicase approach to study governance mechanisms and their impact on the quality of health care supply networks. For the organizations they studied, they did not find formal network structures and suggested that both formal and informal network structures are needed to avoid quality problems [26]. The design and integration of a well-functioning supply chain constitute important work, but there is also a shortcoming; that is, supply chain research mostly examines organizational cooperation from the perspective of production operations, but it is difficult to include the nonproduction nature of diversified organizations within the scope of research.

### 2.2. Emergency Organization Networks

In recent years, scholars have integrated emergency organizations into a network form to address severe crises [11] because the scope and harm of social disasters have continuously expanded. In previous studies, the network of emergency management organizations has been dominated by institutionalized organizations based on emergency plans, regulations and policies. Therefore, the response efficiency of institutionalized networks has received considerable attention [27]. Many studies have examined the main factors, including communication of information [28] and response speed [29], that affect organizational performance in a complex environment. Moreover, the impact of cross-departmental alliances on organizational output, efficiency and flexibility [30], as well as the results of workflow integration [17], are often mentioned. Through the horizontal and vertical integration of emergency organizations [8] and cross-level and cross-jurisdiction cooperation [31], operational capacity can be improved. With the deepening of this research, the rules of network construction have been gradually refined. Scholars have established different connection criteria to shape various emergency organization networks, including policy documents for emergency planning networks and management activities for practice networks [3,15], daily collaboration networks for emergency preparedness [32], emergency cognition and perception networks [33], and other networks constructed in accordance with different structures, resources and types [34]. The pluralistic rules of network construction provide a theoretical basis for promoting a social emergency supply support network.

### 2.3. The Expansion of the Emergency Management Network

When nonprofit organizations, private organizations and even individuals join emergency management, the boundary of the original cooperation system collapses [3]. The fuzzy boundary of the network enables the emergency organization system to be expanded to a more complex cooperative relationship of members. Therefore, research on organizational cooperation networks has shifted from focusing on horizontal and vertical fragmentation to focusing on cross-boundaries, cooperative forms and network effects such as structure and embeddedness [17,35]. The organizational network is established by members and their relationships, and a complex relationship structure and associations are built. The expansion of the organizational network begins from this. Existing studies have shown that structures such as links, bridges and cross-boundary connections [2,36] directly affect the formation and expansion of organizational networks.

In the field of social network analysis, the ERGM is established to explore structural effects. The ERGM uses structural types such as chains, stars and rings as well as relational types such as transfer and sharing to explain the formation mechanism of organizational networks [37,38]. Thus, it provides a feasible research framework for the expansion of organizational networks. Similar studies about emergency management are emerging. Jun et al. identified the dynamic structure of a cross-organizational post-disaster emergency management network and analyzed the influence of the endogenous structural effect and exogenous community attributes on the relationship between organizations [39]. Jingjing et al. tested the impact of the interaction structure and the organizational attributes of the emergency management network in the Wenchuan earthquake and found a significant network structure based on transitivity and homogeneity [40]. Hossain et al. took forest fires as the disposal scenario and studied structures such as stars and triangles and their influence on organizational networks [41]. McAllister et al. introduced the coordination of emergency management networks to explain the network structure and its interaction across scales [42]. As a method for testing the network formation principle, the ERGM has inspired the idea for this research on the expansion of the cooperative networks of supply support organizations in major public health emergencies.

Overall, current research on cooperative networks for emergency management is flourishing. A consensus on the participation of numerous organizations has gradually been reached, motivating abundant research achievements in relevant fields. The social network blurs the original boundary of the system, which is the structural basis for the expansion of emergency supply support cooperation. Emergency supply support requires social participation in emergency management activities [1,43], however, previous studies have neglected the mechanism of network expansion in the development of emergency organization systems. To fill in the gap, this paper constructs three ever-expanding cooperative networks and probes the expansion mechanism based on previous studies to provide a theoretical reference for emergency supply support and emergency response decision-making.

## 3. Research Design

### 3.1. The Research Framework

**Figure 1:**
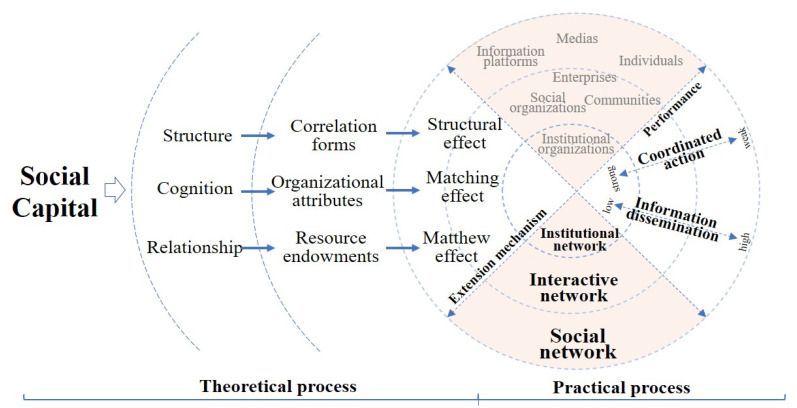
The research framework.

Based on the objectives of this research, we designed a research framework that includes theoretical and practical processes, as shown in Figure 1. The structural effect, matching effect and Matthew effect are used as the joint points to connect the two processes above. This study provides a foundation for research methods.

From the perspective of the theoretical process, whether as a theoretical tool or a practical element, social capital has become an essential component in the field of emergency management [2]. Social capital theory holds that organizational networks can be formed naturally or constructed socially, which is the basis for establishing a network across domains and organizational boundaries. Relationships express the characteristics of occupiers, locations, resources, rules and procedures. The relationship between members reflects interdependence and transferability and relies on reciprocity, trust and other attributes [2,44]. For emergency supply support, the cooperative relationships can improve access to social capital. At the same time, a cooperative relationship is a realistic connotation of the edge of the network, and the local structure formed by the complex edge also reflects the further extension of the relationship. Furthermore, a cooperative relationship can evolve into an expansion trend and effect of the cooperative network for emergency supply support.

From the perspective of the practical process, when organizations participating in emergency supply support gradually expand from governmental institutions to social organizations, enterprises and volunteers, the rules of action gradually accommodate administrative constraints, reciprocal cooperation and voluntary donation [45]. In this process, changes in the structure and types of cooperation can be explained by the performance of information transmission and action coordination [33]. In terms of information transmission, the demand for and dispatching of materials are usually the main content of emergency supply activities, and cyberspace has become a common platform for information acquisition and exchange. With the expansion of cooperative networks, the method of information transmission has changed from administrative channels to Internet platforms. Thus, the timeliness and amount of information have increased, and the fuzziness has been enhanced. The action of emergency supply support mainly relies on institutionalized departments and is supplemented by some regular social resources. The guidance of emergency supply support has changed from command-and-control action to cross-level and cross-jurisdictional network coordination or less compact social cooperation. Exploring the expansion mechanism of networks in the process from institutionalization to socialization is helpful in promoting the integration and complementarity of information transmission and action coordination.

Based on social capital theories [46] and the combination of the social characteristics of emergency supply support and the ERGM, relationships, structures and cognition are regarded as the essential dimensions of organizational cooperation in this paper. A relationship is a direct representation of whether a connection is established between organizations. From the perspective of the overall network, the greater the number of contacts the members establish is, the more obvious the advantages of their network status and the more resources they can influence. Structure refers to the form of association around a node or in the local part of the network. Different structures embedded in the network reflect different action and information transmission paths. Cognition is an endogenous factor of organizations that influences the establishment of cooperative relationships through organizational attributes. Therefore, this paper uses the structural effect, matching effect and Matthew effect to examine the expansion mechanism of cooperative networks of supply support organizations in public health emergencies.

#### 3.1.1. The Structural Effect

One of the main viewpoints of social network analysis is that network edges are interdependent [47]; that is, whether an edge occurs is conditional on the configuration of other edges in the network. The autocorrelation mechanism of edges emerges as a structure at the macro level in the network, thus the potential endogenous network expansion mechanism can be inferred through structural features [37]. Social capital is embedded in the network structure. In a cooperative network in which various emergency supply support organizations participate, the expansion of the network structure can be analyzed in depth by describing indicators such as the number of edges, density, clustering coefficient, structural holes, and other shapes such as K-star, sharing and closed cycles [48].

#### 3.1.2. The Matching Effect

The principle of homogeneity and the “like-me” hypothesis in social capital theory propose that “social interaction tends to occur between similar individuals” [49]. The corresponding phenomenon in the expansion of networks is that nodes with similar attributes are more likely to establish connections, which is called homophily [48]. Existing studies often use the jurisdiction, ownership and affiliation of organizations as indicators to examine the matching tendency to illustrate the key attributes and their impact on relations [40]. In this paper, the critical attributes of the matching tendency can come from the region, industry field and level of the organization based on the activities of emergency supply support. The matching effect can explain the formation of cooperative relationships from the perspective of potential trends, which are one of the driving forces for expansion.

#### 3.1.3. The Matthew Effect

The position in the network affects opportunities to acquire and utilize social capital [50]. The tendency to form network relations is not equal, that is, there is a nonuniform degree distribution. The Matthew effect in the process of network expansion refers to members being more willing to establish cooperative relationships with other members in a dominant position, and it can also be used to indicate whether there is a tendency toward polarization in network expansion. One of the practice goals is to gather emergency social capital through normalized preparedness and to put it into use quickly when an emergency occurs. Members in advantageous positions often have significant emergency resources at their disposal. The Matthew effect is derived from cooperative relationship cognition. In the uncertain environment of an emergency, the establishment of a relationship may depend on attention to the authority and core members.

### 3.2. Data and Methods

#### 3.2.1. Data Source

COVID-19 first appeared in Wuhan, Hubei Province, China, in late 2019 and later developed into a global public health event. Faced with multiple tasks, such as scale differentiation, sudden fluctuations in the epidemic, and the fundamental life needs of residents in isolation, national and local governments have used the application and approval process to organize several enterprises as key producers of materials for epidemic prevention and control to enhance the emergency management capacity of society. A relevant special audit report shows that from the outbreak of the COVID-19 pandemic to 31 August 2020, the Red Cross Society of China received donations worth more than 800 million yuan, including approximately 600 million yuan in funds and 200 million yuan in materials. A total of 42 batches of materials were donated, including drugs, masks, protective clothing, goggles, gloves, nucleic acid detection agents, consumables, and disinfection and sterilization products for daily use [51]. To address the pandemic, more than 30 provinces in China initiated a first-level emergency response, constructing a typical Chinese situation for the study.

Based on web crawler collected a large amount of network data by setting the “epidemic prevention” and “materials” as keywords. (1) The official policy documents from National Health Commission’s official website and the State Council website database were collected, with the acquisition time being from January 2020 to February 2021. (2) Large news and information websites were used to screen the news, and the collection time was January 2020–February 2021. (3) Sina Weibo was used to search institutional microblogs directly related to keywords and their follow lists, and screen key users and their friends.

**Figure 2:**
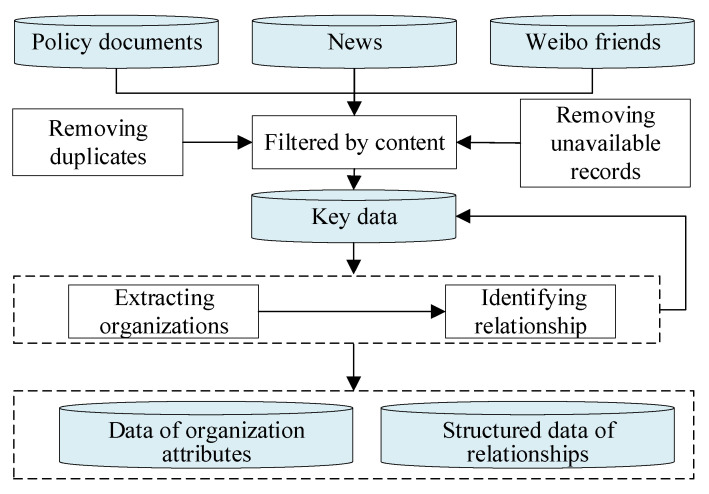
Data processing procedure.

To transform textual data into structured data that can be used to build a network, it is necessary to first identify the subjects in the text and to then identify whether the relationship between the subjects can be established. The processing procedure is shown in Figure 2. The principle here is that we believe that there is a cooperative relationship between subjects implementing unified policy instructions, that subjects in the same news event can form a relationship, and that the attention of Sina Weibo subjects is also a natural relationship. In the process of subject screening and relationship identification, we also excluded materials that were difficult to use to represent the subject relationships. Finally, 140 policy documents, 999 news records and 666 Sina Weibo relationships were used as the research data set.

We identify organizations in the following categories. The emergency supply support organizations in this emergency mainly include national and local government-related organizations that allocate and coordinate emergency supplies in the form of administration and command; social organizations, enterprises, media and other nongovernmental organizations that perform the tasks of material production, circulation, distribution, scheduling and information dissemination in a spontaneous or semi-spontaneous form [48]; and individuals who volunteer, donate, and disseminate information based on their own will. The construction of emergency supply support organization systems and their various representatives is summarized in Table 1.

#### 3.2.2. Research Methods

The ERGM, which is developed based on relationships, provides an important research method for the expansion mechanism of cooperative networks [36,47,52]. The characteristics of the network structure, network members and the relationship pattern of social participation can be explored by logistic regression considering dependency relationships [37,53,54]. With the emergency supply support organization as the network node, the association between organizations through various forms and channels can be identified as a cooperative relationship, which forms the link and network structure among the nodes. Thus, to obtain the effect of each factor in the process of network expansion, a model can be built to estimate the network structure and node attributes of organizations.

The formula is as follows:

(1) $$P\left(Y=y\right)=\left(\frac{1}{k}\right)e^{\sum_{A}\eta_{A}g_{A}\left(y\right)}$$

where the parameter *k* can ensure that network structure expansion has a probability range of 0~1. *Y* represents the matrix network, and y is the observation value of the matrix network. A includes all network structures, while *η_A_* represents the parameters associated with *A*. If and only if the variables in *A* are conditionally independent does *η_A_* = 0. Additionally, *g_A_*(*y*) is the network statistic related to *A*. If the structure in A can be observed in network y, then *g_A_*(*y*) = 1; otherwise, *g_A_*(*y*) = 0.

The construction of the ERGM starts from a simple stochastic graph model, namely, a null model. The null model usually only investigates the edge index of the network. Then, the attributes of the members for emergency supply support are incorporated into the model by adding main effects and interactive statistics, generating a binary independent model. Finally, the main effects of geometric statistics and the complement of interaction statistics are incorporated into the model. Variables are proposed based on the research framework and research methods, as shown in Table 2.

## 4. Results

### 4.1. Network Characteristics

**Figure 3:**
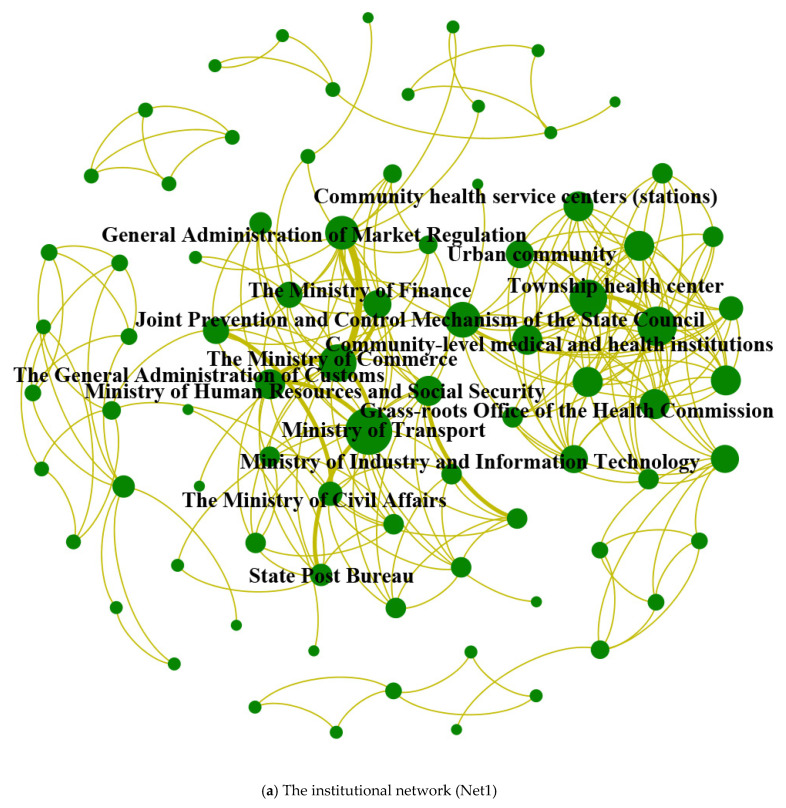

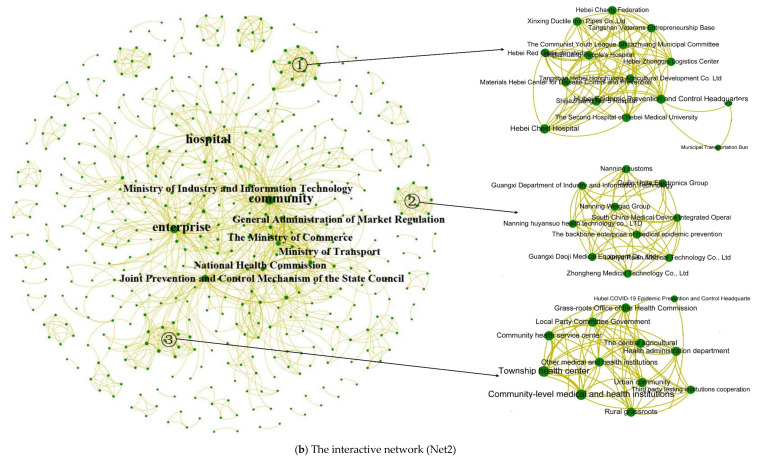

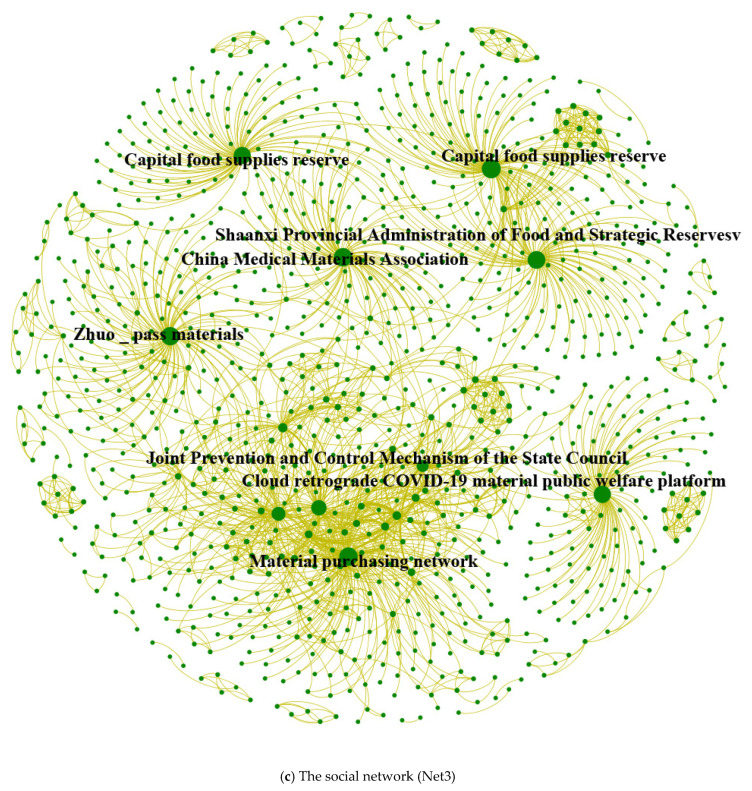
Cooperative Networks of Supply Support Organizations.

Three networks were constructed to examine the expansion mechanism. First, the institutional network of emergency supply support (Net1) is built based on the website of the National Health Commission and the policy database of the State Council. The interactive network (Net2) consists of Net1 and the organization are filtered from information from news websites. Finally, the social network (Net3) is composed of Net2 and organizations from Sina Weibo data. Gephi software (Bastian, Heymann, Jacomy and Gephi. Compiègne, France) [55] is used for visualization and the three networks are drawn in Figure 3 with some detail expansions.

Figure 3 presents the shapes and sizes of three networks as well as some important organizations. The key nodes in the institutional network (Net1) include the Ministry of Transport, grassroots medical and health institutions, township and township hospitals, the Ministry of Commerce, the joint prevention and control mechanism of the State Council, the General Administration of Market Regulation, the Central Agricultural Office, rural grassroots units, and urban communities. The key nodes in the interactive network (Net2) include communities, enterprises, the Ministry of Transport, the Ministry of Commerce, villages, the joint prevention and control mechanism of the State Council, township health centers, and grassroots medical and health institutions. The key nodes in the social network (Net3) include the China Medical Supplies Association, the cloud-retrograde COVID-19 material public welfare platform, the material reserve network and various media organizations. Since the scale obviously expands, the three networks can express the trend of network morphology expansion and facilitate research on the expansion mechanism.

The organizations provided by the policy database and the official website of the government can be distinguished into diverse types. The membership in the institutional network (Net1) and the interactive network (Net2) are counted to obtain the proportion of types, as shown in Table 3.

Table 3 shows that the proportion of organizational types between the institutional network (Net1) and the interactive network (Net2) has significantly changed. National administrative institutions, local administrative institutions and enterprises constitute the main cooperation organizations for emergency supply support, accounting for 64% and 71% of the organizations in the institutional network (Net1) and the interactive network (Net2), respectively. However, different proportions of these three organizational types are revealed by comparing the two networks. In the institutional network (Net1), national administrative institutions and local administrative institutions account for more than 50%, while in the interactive network (Net2), enterprise organizations account for more than 30%. Thus, in the interactive network (Net2), social enterprises have become the most common type of organization. In addition, the proportions of trade associations, health care-related institutions, public institutions, grassroots units and other types of organizations in the two networks are similar.

Indicators of overall network characteristics parameters are calculated, as shown in Table 4.

The results in Table 4 imply that organizations connect relatively loosely in the entire network of emergency supply support. The density index is used to examine the closeness of the connection between nodes in the network. The greater the network density is, the closer the connection between nodes, the greater the influence of the network on the behavior and attitude of member organizations, and the more resources it may provide. The densities in the three networks are all lower than 0.1, indicating that the network connection is weak. This indicates that the direct requirement for the emergency supply network should be strengthened. For the three networks, the parameters related to network size, such as diameter and average path length, all increase with the expansion of the cooperative organization system. However, the parameters of network efficiency, such as network density, the average degree and the clustering coefficient, tend to decrease. This implies that from the institutional network (Net1) to the interactive network (Net2) to the social network (Net3), a larger organizational system and network scale form with types of enterprises, social organizations and other organizations joining continually, but it does not improve the network efficiency.

The network characteristic parameters reflect the different transmission mechanisms of the three networks through further analysis. The institutional network (Net1) with a larger average degree and a smaller average path length indicates that the construction of governmental institutions and departments provides mandatory guarantees for emergency supplies. The clustering coefficient in the interactive network (Net2) is the largest and three typical cluster structures (①, ② and ③) are displayed as detail amplifications in Figure 3b. The above indicate that the efficiency of the interactive network (Net2) is at the middle level among the three networks; however, the connection of local cliques is more obvious. In social networks (Net3), the organizations provided by social platforms account for a large proportion. However, the clustering coefficient and average degree are low, with a prominent status of core nodes. The topic of supply shortages can cause great information dissemination on social platforms. It is inferred that there is a lack of institutional nodes whose core tasks involve information related to supply guarantees and that information transmission still needs to be completed through the help of core nodes.

Structure is the skeleton formed by the network, and the number of each structure is statistically shown in Table 5.

**Figure 4:**
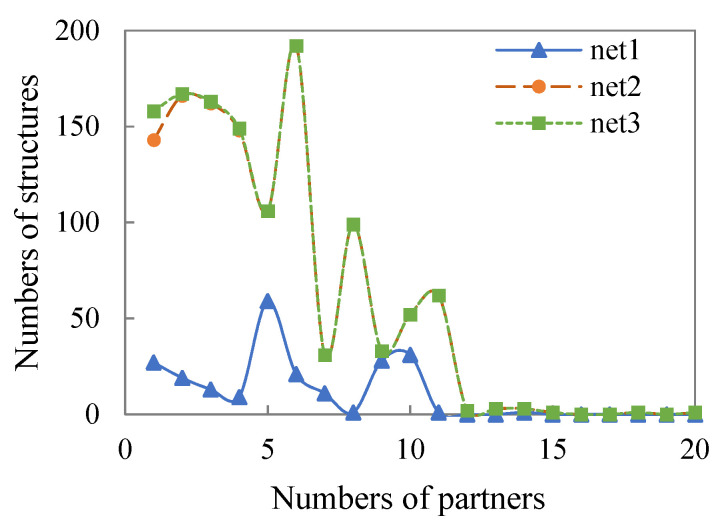
The number of DSP (dyadwise shared partners).

**Figure 5:**
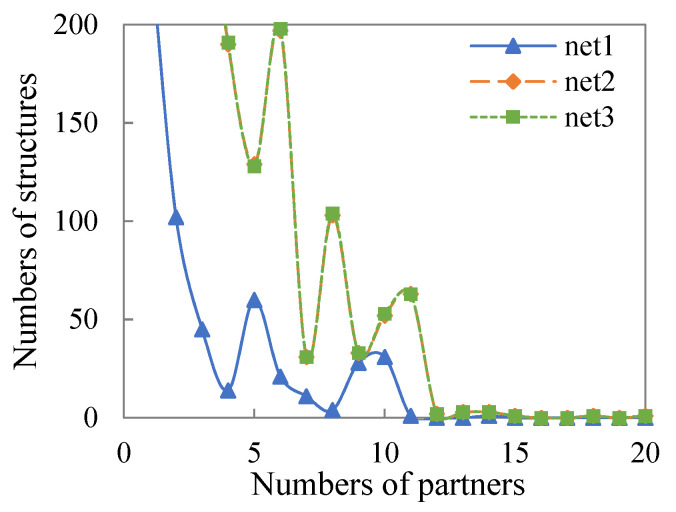
The number of GWESP (geometrically weighted edgewise shared partners).

In general, in contrast to the institutional network (Net1), the number of various structures in the interactive network (Net2) has increased significantly, while the structures in the social network (Net3) have not improved significantly compared with the interactive network (Net2). Based on the distribution of structures shown in Table 5, although the number of edges expands smoothly, other complex structures show obvious differences between the institutional network (Net1) and the interactive network (Net2). At the same time, as shown in the distribution of shared structures in Figure 4 and Figure 5, the difference between the institutional network (Net1) and the interactive network (Net2) is more obvious, while there are very few increases in the social network (Net3). The above shows that the social enterprises and organizations integrated into the interactive network (Net2) play an important role in the formation of the complex relationship structure and reflect better structural embeddedness and network functions.

### 4.2. Model Estimations

The statnet tool in R software (R Development Core Team, Vienna, Austria) was used, and Monte Carlo maximum likelihood estimation (MLE) results are shown in Table 6, Table 7 and Table 8. The Akaike information criterion (AIC) and the Bayesian information criterion (BIC) are the fit indexes of the information criteria; the smaller their values in the unified model are, the better the fit result.

Table 6, Table 7 and Table 8 respectively show the ERGM results of institutional network, interactive network and social network. For each network, estimates of four models were given. Then we analyze the expansion mechanism in terms of three effects.

#### 4.2.1. Analysis of the Structural Effect

The estimation results show that the cooperative relationship of emergency supply support is relatively loose. The null model can be used to analyze the expansion situation based on the cooperative relationships in the organizational network. The negative coefficient in the null model indicates that the network density is less than 50% [52]. Therefore, there are no tight cooperative relationships in any of the three networks. The nature of K-stars reflects the expansion trend of the node relationship. However, most of the parameters of these variables in the estimated results are negative, indicating that the nodes do not trend to increase relationships and that there is no tendency of structural expansion in the cooperative network of emergency supply support organizations. Therefore, it is difficult for the organizational network of emergency supplies in China to expand by promoting the formation of a complex relationship structure.

#### 4.2.2. Analysis of the Matching Effect

The matching effect is significant in the interactive network (Net2), which demonstrates that type matching contributes to expanding the cooperative network of emergency supply support organizations. In this paper, the institutional network (Net1) and the interactive network (Net2) are selected to conduct the matching test. The estimators of the matching effect are not significant (0.136, *p* = 0.285) in the institutional network (Net1) but are significant (0.118, *p* = 0.040) in the interactive network (Net2). This illustrates that organizations of the same type are more inclined to establish cooperative relationships in the interactive network. However, the institutional network does not confirm this rule. This reflects the insufficiency of horizontal cross-organizational connections in governmental departments. The interactive network (Net2) consists of government agencies, enterprises and social organizations that carry out the entire process from upstream production to downstream distribution. It is possible that there is a close relationship between homogeneous types of social organizations and between homogeneous types of governmental institutions but not between the two types of members.

#### 4.2.3. Analysis of the Matthew Effect

In general, the Matthew effect, which takes the node degree as an indicator, plays no obvious role in the network expansion of emergency supply support organizations, but the local connection tendency plays a significant role. The degree and clustering coefficient are selected as the basic indexes for the estimation of the Matthew effect in this paper. The results using the degree as an indicator show that the Matthew effect is not significant in the three networks, although it is slightly significant in the institutional network (Net1), having a low value (−0.027, *p* = 0.035). The estimators using the clustering coefficient as the index are significant in both the interactive network (Net2) and the social network (Net3) (0.158, *p* < 0.000; −0.078, *p* < 0.000). The clustering coefficient of a node quantifies the degree to which its neighboring nodes cluster together to form local connections. Based on the above analysis, there is no obvious tendency of network expansion with node degree as an indicator, while the connection in local cliques can be strengthened after social enterprise organizations and social platform organizations are joined.

## 5. Discussion

Effective and efficient emergency supplies are of great value in relieving social pressure and maintaining social stability in a crisis. With the active participation of government institutions, enterprises, other social organizations and the public in solving public affairs, an emergency organization action system is composed of numerous participants. Social capital can also compensate for deficiencies in government management. Based on the developing requirements of supply support cooperation in public health emergencies from institutionalization to socialization, this paper constructed three networks: an institutional network, an interactive network and a social network. The expansion mechanism of the organizational network was revealed by considering the network characteristics.

The network characteristics indicate that the cooperative network of emergency supply support organizations in China is relatively loose. Comparing the interactive network with the institutional network, this study finds that the composition of organizational types is significantly different and that social enterprises have become an essential constituent for emergency supply support systems. This study also finds that the institutional network established by policy norms is relatively balanced, with the highest coordination efficiency from the perspective of network structure. This result is closely related to the emergency response rules and drills conducted in governmental organizations, which provide institutional guarantees for national emergency management. However, the socialization of the institutional network remains weak. After forming the interactive network and the social network, there are more obvious features of block factions and core nodes. The importance of network attributes such as the relationship structure and member position begin to manifest. The interactive network, with increased enterprise and social organization, facilitates more complex relationship structures, but the social network with organizations from social platforms has not yet increased the complex relationship structures. In conclusion, the addition of diversified social organizations and enterprises has greatly changed the cooperative network structure of emergency supply support organizations, but the expansion of networks has not distinctly promoted efficiency.

Our analysis of the expansion mechanism shows that it is difficult for the current network to expand through an increase in various complex relationship structures. The interactive network embodies a distinct matching effect and Matthew effect, indicating that the network expands after the extensive participation of diversified enterprises and social organizations. First, the same types of organizations tend to develop cooperative relationships. Then, the Matthew effect shows that the members in the dominant position of the network have strong attraction and are more inclined to establish connections with other members. Thus, two ways to expand the cooperative network of emergency supply support organizations are indicated. In addition, although the formation of a complex relationship structure has difficulty providing an obvious extension to the network, there has been a trend of strengthening the small group structure. Therefore, network expansion can also be facilitated by the increasing cooperation of emergency supply support organizations in minor factions.

The study case in this paper describes the situation in China, and some of the results are characteristic of the typical Chinese scenario. However, since the COVID-19 outbreak has become a global pandemic crisis, a more general discussion will take place. In a public health emergency, market failure is very likely to occur [56]. The outbreak has caused a decline in productive capacity. Widespread participation helps to scale up the production of materials, and strong command and oversight can ensure that materials go where they are needed rather than where there is greater exchange capacity, which corresponds to the original intention of studying the cooperative network of emergency supply support: first, to increase the accumulation of emergency social capital and, second, to enhance the effectiveness and availability of emergency social capital.

Compared with existing studies, our study consisted of several different concerns. The existing research includes institutional network, planned network, actual network, communication network and even risk perception network. Such studies tend to focus on and compare the performance of networks. By adopting a progressive perspective from institutionalization to socialization, we developed three cooperative networks of institutional, interactive and social emergency supply support organizations. The research method of ERGM gives us a relatively complete and straightforward framework, which is of great help to the study of network mechanism. In various studies, different structures and embeddedness have been studied. According to the research results of the existing literature, the density of cooperation networks formed by emergency organizations is generally not high, and the cooperation tends to be matched. The Matthew effect has not yet been tested in emergency management networks, and we have closely examined the effect of bars on a broad network. But it may exist in more liberal networks, such as entirely social networks, collaborative networks of innovation. Our study is expected to establish a broader emergency supply base, so that the Matthew effect may be better reflected in the future.

This paper is an important attempt to research the cooperative network of emergency supply support. One possible limitation may stem from the processing of the data obtained from the sources used in this study. Although the data obtained from the sources used in this study have also been used in previous studies, we are still aware that some standards for data construction and screening are not perfect in the process of implementation. Relatively explicit processes are also established to reduce their impact on the results, but there may still be some bias. However, they mainly affect the absolute scale of the organizational network, and previous studies have demonstrated the feasibility of research based on such data.

## 6. Conclusions

Supply support is a vital task for dealing with major public emergencies. Seeking the extensive integration of socialized organizations is a requirement for improving practical ability based on governmental departments maintaining reliable action power. In the face of the demand to socially expand emergency supply support, this paper establishes a research framework that includes three successively increasing networks. Using the ERGM, this paper proposes the theoretical expansion mechanism of cooperative networks based on the structural effect, matching effect and Matthew effect. Our results can enrich theoretical references for the government and society to develop strategies for emergency supply support.

Our results show that the parameters related to the network scale, such as diameter and the average path length, all increase with the expansion of the scale of the cooperative organization system for emergency supply support. However, the parameters closely related to network efficiency, such as network density, the average degree and the clustering coefficient, show a decreasing trend. Compared with the institutional network, the number of shared structures in the interactive network dominated by social enterprises increases. The analysis of network characteristics implies that the three networks increase in order and that the complex relationship structure increases; however, but network efficiency does not improve. The ERGM results show that the matching effect and the Matthew effect, which take the type as the index, influence network expansion. The structural effect is consistent with the parameter expression of network characteristics.

Cooperative relationships are the theoretical basis of this paper and the essential element for forming social networks. However, in some fields, the strength of a relationship can be used to measure social capital. This paper takes the network structure characteristics and network expansion as the research objective and weakens the influence of relationship strength to some extent. There may be differences in the social capital stock among different cooperative networks of emergency supply support organizations. In other words, the same change in density in the institutional network and the social network may affect different social capital. This issue will constitute an important line of research.

## Figures and Tables

**Table 1:** Organizational system of supply support in public health emergencies.

Type	Task	Representative
National administrative institutions	Comprehension of the overall situation and the allocation and coordination of national emergency resources	The Joint Prevention and Control Mechanism of the State Council, the Ministry of Industry and Information Technology, the National Health Commission, the Ministry of Transport, etc.
Local administrative institutions	Organization, deployment and coordination of local emergency supplies	Municipal governments, the National Development and Reform Commission, the Headquarters for Epidemic Prevention and Control, the National Health Commission, the Market Supervision and Administration, etc.
Trade association organizations	Material collection, distribution, docking and deployment assistance	The Red Cross Society of China, the Guangxi Overseas Chinese Compassion Foundation, the Hebei Charity Federation, the Hubei Zhuoer Public Welfare Foundation, the China Life Care Association, the Medical Device Industry Association, etc.
Public institutions	Material collection, distribution, docking and deployment assistance	Public schools of all kinds and other institutions
Social enterprises	Production and circulation of emergency supplies	Chengdu Yute Logistics Co., Ltd., Dongguan Kuaiyuda Automation Equipment Co., Ltd., Guangxi Daoji Medical Equipment Co., Ltd., China Resources Vanguard, Sunway Pharmacy, etc.
Health care-related institutions	Efficient and effective collection and usage of emergency medical supplies	Hospitals, community health service centers, isolation hospitals, etc.
Mass organizations and communities	Distribution of materials and other auxiliary work	Communities (neighborhood committees), villages (village committees), etc.
Individuals and other organizations	Voluntary activities, individual donations, information promotion, etc.	Citizens and other Sina Weibo users, etc.

**Table 2:** Variables and their explanations.

Effects	Variables	Diagrams	Explanations
Cooperative relationship	Edges	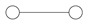	The basic effect of connect between organizations
Structure effect	Three trail	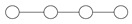	Single line structure
	K-stars	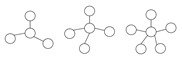	Relationship structure of core members
	Cycle(k)	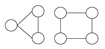	Cyclical closed structure
	GWESP/DSP	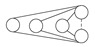	Shared structure
Matching effect	Nodematch	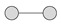	The tendency of members with the same attributes to form cooperative relationships
Matthew effect	Nodecov	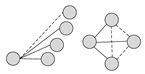	Global or local core node connection advantage and node position advantage polarization tendency

The gray nodes indicates that some attributes and indicators may be involved. GWESP: geometrically weighted edgewise shared partners. DSP: dyadwise shared partners.

**Table 3:** Organizational types for emergency supply support and their proportions.

Types	Net1	Net2
Local administrative institutions	10%	20%
National administrative institutions	46%	18%
Social enterprises	8%	33%
Trade association organizations	7%	7%
Health care-related institutions	10%	3%
Mass organizations and communities	8%	9%
Public institutions	7%	4%
Others	3%	6%

**Table 4:** Network characteristic parameters.

Items	Net1	Net2	Net3
Density	0.068	0.010	0.003
Average degree	6.072	5.143	3.446
Diameter	6	8	10
Average clustering coefficient	0.869	0.882	0.823
Mean path length	2.879	3.569	4.802

**Table 5:** Numbers of network structures.

Structures	Numbers in Net1	Numbers in Net2	Numbers in Net3
Edges	229	1277	1941
3-trial	14,964	162,605	184,645
3-stars	9	80	82
4-stars	9	58	59
5-stars	4	45	45
6-stars	13	26	26
7-stars	2	41	41
Cycle(3)	411	1967	1975
Cycle(4)	2064	9415	9510

**Table 6:** Results of the institutional network (Net1).

Variables	Model 1-1	Model 1-2	Model 1-3	Model 1-4
Edges	−2.629 *** (0.000)	−2.634 *** (0.000)	−2.792 *** (0.000)	−2.747 *** (0.000)
4 stars	-	−0.958 * (0.008)	-	-0.918 * (0.011)
5 stars	-	−2.146 *** (0.000)	-	−2.125 *** (0.000)
6 stars	-	−0.629 * (0.042)	-	−0.580 (0.064)
7 stars	-	−2.207 *** (0.000)	-	−2.191 *** (0.000)
Nodecov (cluster)	-	-	0.251 (0.079)	0.16241 (0.166)
Nodecov (degree)	-	-	−0.027 * (0.035)	−0.018 (0.087)
Nodematch (type)	-	-	0.165 (0.249)	0.136 (0.285)
AIC	1680	1647	1648	1648
BIC	1686	1677	1685	1697

The *p* values are in parentheses. * is *p* < 0.05 and *** is *p* < 0.001. AIC is the Akaike information criterion; BIC is the Bayesian information criterion.

**Table 7:** Results of the interactive network (Net2).

Variables	Model 2-1	Model 2-2	Model 2-3	Model 2-4
Edges	−4.559 *** (0.000)	−4.520 *** (0.000)	−5.016 *** (0.000)	−4.834 *** (0.000)
4-stars	-	−0.877 *** (0.000)	-	−0.857 *** (0.000)
5-stars	-	−1.176 *** (0.000)	-	−1.158 *** (0.000)
6-stars	-	−1.589 *** (0.000)	-	−1.576 *** (0.000)
7-stars	-	−0.843 *** (0.000)	-	−0.839 *** (0.000)
Nodecov (cluster)	-	-	0.244 *** (0.000)	0.158 *** (0.000)
Nodecov (degree)	-	-	0.004 (0.234)	0.002 (0.382)
Nodematch (type)	-	-	0.128 * (0.025)	0.118 * (0.040)
AIC	14,214	14,064	14,192	14,052
BIC	14,224	14,113	14,231	14,129

The *p* values are in parentheses. * is *p* < 0.05 and *** is *p* < 0.001. AIC is the Akaike information criterion; BIC is the Bayesian information criterion.

**Table 8:** Results of the social network (Net3).

Variables	Model 3-1	Model 3-2	Model 3-3	Model 3-4
Edges	−5.684 *** (0.000)	−5.519 *** (0.000)	−5.698 *** (0.000)	−5.503 *** (0.000)
3 stars	-	−1.841 *** (0.000)	-	−1.8012 *** (0.000)
4 stars	-	−2.150 *** (0.000)	-	−2.097 *** (0.000)
5 stars	-	2.1901 *** (0.000)	-	−2.1167 *** (0.000)
6 stars	-	−2.289 *** (0.000)	-	−2.278 *** (0.000)
Nodecov (cluster)	-	-	−0.137 *** (0.000)	−0.078 *** (0.009)
Nodecov (degree)	-	-	0.000 (0.864)	0.000 (0.752)
AIC	26,353	25,411	26,343	25,433
BIC	26,365	25,468	26,377	25,513

The *p* values are in parentheses. *** is *p* < 0.001. AIC is the Akaike information criterion; BIC is the Bayesian information criterion.

## Data Availability

Data sharing not applicable.
